# Smoking Cessation Among Gender Minority Populations, Cis-women, and Cis-men: Findings From the International Tobacco Control Netherlands Survey

**DOI:** 10.1093/ntr/ntac283

**Published:** 2022-12-13

**Authors:** Gera E Nagelhout, Nikita L Poole, Cloé Geboers, Tessa Magnée, Anne Marije Kaag, Floor A van den Brand, Bas van den Putte, Hein de Vries, Geoffrey T Fong, Marc C Willemsen

**Affiliations:** Department of Health Promotion, Maastricht University (CAPHRI), Maastricht, The Netherlands; IVO Research Institute, The Hague, The Netherlands; Department of Health Promotion, Maastricht University (CAPHRI), Maastricht, The Netherlands; IVO Research Institute, The Hague, The Netherlands; Department of Health Promotion, Maastricht University (CAPHRI), Maastricht, The Netherlands; The Netherlands Expertise Centre for Tobacco Control, Trimbos Institute, Utrecht, The Netherlands; Erasmus School of Social and Behavioural Sciences, Erasmus University Rotterdam, Rotterdam, The Netherlands; Department of Clinical, Neuro and Developmental Psychology, VU Amsterdam, Amsterdam, The Netherlands; Department of Family Medicine, Maastricht University (CAPHRI), Maastricht, The Netherlands; Department of Communication, University of Amsterdam (ASCoR), Amsterdam, The Netherlands; Department of Health Promotion, Maastricht University (CAPHRI), Maastricht, The Netherlands; Department of Psychology, University of Waterloo, Waterloo, ON, Canada; School of Public Health Sciences, University of Waterloo, Waterloo, ON, Canada; Ontario Institute of Cancer Research, Toronto, ON, Canada; Department of Health Promotion, Maastricht University (CAPHRI), Maastricht, The Netherlands; The Netherlands Expertise Centre for Tobacco Control, Trimbos Institute, Utrecht, The Netherlands

## Abstract

**Introduction:**

Little is known about smoking cessation among gender minority populations compared to cisgender individuals (whose gender matches their sex assigned at birth). We examined differences between smokers from gender minority populations, cis-women, and cis-men in the heaviness of smoking, quit intentions, use of cessation assistance, quit attempts (ever tried and number), and triggers for thinking about quitting.

**Aims and Methods:**

We used cross-sectional data from the 2020 International Tobacco Control Netherlands Survey. Among smoking respondents, we distinguished (1) cis-women (female sex, identified as women, and having feminine gender roles; *n* = 670), (2) cis-men (male sex, identified as men, and having masculine gender roles; *n* = 897), and (3) gender minorities (individuals who were intersex, who identified as nonbinary, genderqueer, had a sex/gender identity not listed, whose gender roles were not feminine or masculine, or whose gender identity and/or roles were not congruent with sex assigned at birth; *n* = 220).

**Results:**

Although gender minorities did not differ from cis-women and cis-men in the heaviness of smoking, plans to quit smoking, and quit attempts, they were significantly more likely to use cessation assistance (20% in the past 6 months) than cis-women (12%) and cis-men (9%). Gender minorities were also significantly more likely to report several triggers for thinking about quitting smoking, for example, quit advice from a doctor, an anti-smoking message/campaign, and the availability of a telephone helpline.

**Conclusion:**

Despite equal levels of quit attempts and heaviness of smoking, gender minority smokers make more use of smoking assistance, and respond stronger to triggers for thinking about quitting smoking.

**Implications:**

Smoking cessation counselors should be sensitive to the stressors that individuals from any minority population face, such as stigmatization, discrimination, and loneliness, and should educate their smoking clients on effective coping mechanisms to prevent relapse into smoking after they experience these stressors. Developing tailored smoking cessation programs or campaigns specifically for gender minority populations can also be useful. Based on the results of our subgroup analyses, programs or campaigns for younger gender minority smokers could focus on the availability of telephone helplines and on how friends and family think about their smoking behavior.

## Introduction

An increasing number of studies examine gender differences in smoking cessation. However, most studies only examine differences between cisgender women and men. Much less is known about smoking cessation among gender minority populations.^1,2^ In this paper, we examine both gender *identity* minority populations and gender *role* minority populations.

First, in this paper gender identity minority populations consist of individuals whose *gender identity* is not congruent with the sex assigned at birth, for example, individuals who identify as nonbinary, genderqueer, or transgender. For cisgender individuals, their gender identity is the same as their sex assigned at birth. There are indications that smoking prevalence is two to four times higher in gender minority populations than in cisgender individuals.^3–5^ Underlying mechanisms of higher smoking prevalence among gender minorities may be stress caused by stigmatization, discrimination, and violence against this group,^6^ but it may additionally be the result of targeted marketing by the tobacco industry.^7^

Second, in this paper gender role minority populations consist of people whose *gender roles* are not congruent with the sex assigned at birth. Gender role conflict is defined in previous literature as a psychological state in which socialized gender roles have negative consequences for the person or others.^8^ It occurs when rigid, sexist, or restrictive gender roles result in personal restriction, devaluation, or violation of others or oneself. Studies have shown that gender role conflict may increase the odds of mental health issues, stress, and problematic substance use.^8–11^ To the best of our knowledge, a possible relation between gender roles or gender role conflict and smoking cessation has not been examined yet.

Intersectionality theory posits that multiple social categories, including gender identity, gender roles, sexual orientation, and race/ethnicity, intersect at both the individual level and at the macro level to reflect systems of privilege and oppression.^12^ Both gender identity minority populations and gender role minority populations face stigmatization and inequalities due to cisnormativity and restrictive gender roles, while some of them also experience other types of systemic discrimination (eg, racism or classism).^13^ The minority stress theory adds that health inequalities are a result of stressors experienced disproportionally by minority groups.^14,15^ Among gender minorities, these include distal stressors, such as harassment, discrimination, and violence, and proximal stressors, such as expectations of rejection, concealment, and internalized transphobia. These stressors together produce poorer mental and physical health outcomes, which include increased use of drugs^14^ and may include more difficulty with smoking cessation.

Besides the importance of examining smoking cessation among gender minority populations, it remains important to examine differences in smoking cessation between cis-women and cis-men. Cis-women experience health disadvantages due to gender inequality, including unequal power relationships, and traditional gender roles that decrease education and paid employment opportunities for women.^10,16^ Cis-women may also experience adverse mental health outcomes, stress, and violence due to their unequal position in society.^17^ There is clear empirical evidence that women have more difficulty with maintaining long-term smoking abstinence than men.^18^ Experience of negative mood and stressful events play a greater role in smoking among women, whilst for men, nicotine dependence, external factors, and a good mood are more often reported triggers for relapse and barriers to cessation.^19–21^ Women report stronger cigarette cravings than men^22^ and have a shorter latency to smoking following stress exposure.^23^ Women also have greater difficulty inhibiting smoking after being exposed to nicotine cues.^24^ There are also biological differences between cis-women and cis-men that may explain women’s difficulties with smoking cessation. Women metabolize nicotine faster than men, especially when using oral contraceptives,^25^ suggests that women may be typically more (physically) dependent on nicotine than men. Despite this, for women, behavioral dependence on smoking is more influential than the nicotine dependence.^19^

The first aim of this study is to examine differences between gender minority populations, cis-women, and cis-men in the heaviness of smoking, quit intentions, use of cessation assistance, quit attempts (ever tried and number), and triggers for thinking about quitting smoking. Furthermore, knowledge about subgroup differences may help to optimize targeted smoking cessation interventions. Therefore, the second aim is to examine interactions of gender with age group and socioeconomic background (education and income).

## Methods

### Setting

A nationally representative cross-sectional survey of smokers was conducted in the Netherlands between September and November 2020, as part of the International Tobacco Control (ITC) Netherlands Survey. In 2020, a 5.1 percentage point gap in smoking prevalence existed between women (17.7%) and men (22.8%),^26^ but the smoking prevalence for gender minority populations in the Netherlands was not known.

### Sample

All 1823 included respondents in the ITC Netherlands Survey in 2020 were currently smoking tobacco at least monthly, were aged 18 years or older, lived in the Netherlands, and had smoked at least 100 factory-made or roll-your-own cigarettes in their lives. Survey data were collected through the internet by Kantar Public, a market research company. They drew quota samples from their large probability-based web panel with adults from the general population of the Netherlands who are willing to participate in survey research. With these quotas, they created a sample of respondents that were representative of adult smokers from the Netherlands with respect to sex, region, and age group (based on data from Statistics Netherlands). Respondents were reimbursed with points that they could exchange for gift cards. Young adults (18–24 years old) were given extra points, worth 5 euros, to encourage participation in this age group. Additional details on the methods are available from the ITC Netherlands Technical Report.^27^

### Measures

The ITC Netherlands Survey was cleared for ethics by the Office of Research Ethics of the University of Waterloo in Canada (ORE#41704).

#### Sex and Gender

Respondents’ sex was measured with the question “What sex were you assigned at birth, meaning on your original birth certificate?” with response options female, male, intersex, sex not listed here (specify), don’t want to say, and don’t know.

Gender identity was examined with the question “What is your current gender identity?” with response options woman, man, nonbinary, genderqueer, a gender not listed here (specify), don’t want to say, and don’t know.

Gender roles were examined with the six-item Traditional Masculinity-Femininity (TMF) scale.^28^ The six items could be rated on a scale from very masculine (1) to very feminine (7) and the items were: I consider myself as…; Ideally, I would like to be…; Traditionally, my interest would be considered as…; Traditionally, my attitudes and beliefs would be considered as…; Traditionally, my behavior would be considered as…; and Traditionally, my outer appearance would be considered as… The reliability of the TMF was very high (Cronbach’s alpha = 0.99). Responses on the six items were averaged to create one TMF score for each respondent.

When we combined the variable that measures sex with the variable that measures gender identity, only 14 out of 1787 respondents could be considered part of a gender identity minority population. Because this sample size was too small for analyses, we combined the gender identity and gender role minority participants to create one gender minority group. While not optimal, this combination can be justified on the basis that individuals with gender roles that do not match their sex assigned at birth or gender identity may experience gender role conflict^8,29^ and both gender identity minority populations and gender role minority populations may experience minority stress,^4,9–11^ although we cannot be sure that all of these individuals see themselves as part of a gender minority population. We created one combined measure that distinguishes between individuals who: (1) had female sex assigned at birth, identified as women, and had feminine gender roles (TMF scale 5–7, referred to as cis-women, *n* = 670), (2) had male sex assigned at birth, identified as men, and had masculine gender roles (TMF scale 1–3, referred to as cis-men, *n* = 897), and (3) individuals who were intersex, or had sex not listed, who identified as nonbinary, genderqueer, or a gender not listed, whose gender roles were not feminine or masculine, or whose gender identity and/or gender roles are not congruent with sex assigned at birth (referred to as gender minorities, *n* = 220). Respondents who answered don’t want to say or don’t know on any of the questions (*n* = 36) were left out of this combined measure and all analyses in this paper. There can be different reasons why respondents would answer don’t want to say or don’t know questions about sex and gender identity/roles. One possibility is that they did not like or understand these personal questions, which is in line with the finding that these respondents had a higher likelihood of answering don’t want to say or don’t know on the income question. Another possibility is that their gender identity is more fluid than our questionnaire allowed. Therefore, we performed sensitivity analyses in which we included the respondents who answered don’t want to say or don’t know as part of the gender minority group. Results from these sensitivity analyses were very comparable with the results from our main analyses in which this group was excluded.

#### Other Demographic Characteristics

Respondents were asked to report their age, educational level, and income level. Age was divided into four categories: (1) 18–24 years, (2) 25–39 years, (3) 39–54 years, and (4) 55 years and older. Educational level was divided into three categories: (1) low (primary and lower prevocational secondary education), (2) moderate (middle prevocational secondary education and secondary vocational education), and (3) high (senior general secondary education, [pre-]university education and higher professional education). Monthly gross household income was divided into four categories: (1) low (≤2000 euros), (2) moderate (between 2000 and 3000 euros), (3) high (≥3000 euros), and (4) not stated (don’t want to say or don’t know).

#### Outcomes

The Heaviness of Smoking Index is an indicator of the level of nicotine dependence and is constructed from the number of cigarettes smoked per day and the time before smoking the first cigarette of the day.^30^ The Heaviness of Smoking Index ranges from 0 to 6, with a higher score indicating a stronger nicotine dependence.

To measure quit intentions, respondents could answer on a four-point scale whether they were planning to quit smoking “within the next month,” “between 1 and 6 months,” “beyond 6 months,” or “not planning to quit.” We dichotomized this variable into whether respondents were planning to quit smoking within the next 6 months or not.

We asked respondents who self-reported to have made a “serious quit attempt” in the last 6 months whether they had used any of the following products and services as part of their last quit attempt: nicotine replacement therapy, stop-smoking medications (eg, varenicline, bupropion), behavioral support, self-help materials, e-cigarettes, and heated tobacco products. We dichotomized this variable into whether respondents used any kind of cessation assistance in the past 6 months or not.

Quit attempts were measured by asking respondents whether they ever tried to quit smoking (yes or no). The number of serious quit attempts was measured by asking respondents who ever tried to quit smoking how many serious quit attempts they ever made. A serious quit attempt was not predefined in the survey and respondents could thus define themselves as how many of their quit attempts were “serious.” We categorized responses into: One attempt, two attempts, three attempts, and four attempts or more. We excluded respondents who ever tried to quit smoking but performed zero serious quit attempts (five respondents).

Triggers for thinking about quitting smoking were examined with the question “In the past 6 months, have each of the following things led you to think about quitting?” This was followed by a list with 15 possible triggers: (1) Concern for your personal health, (2) Concern about the effect of your cigarette smoke on nonsmokers, (3) Society’s disapproval of smoking, (4) The price of cigarettes, (5) Smoking restrictions at work, (6) Smoking restrictions in public places like restaurants, cafés, and pubs, (7) An anti-smoking message or campaign, (8) Availability of a telephone helpline or information line, (9) Advice from a doctor, or other health professional to quit, (10) Free, or lower cost, stop-smoking medication, (11) Warning labels on cigarette packages, (12) Setting an example for children, (13) Close friends and family’s disapproval of your smoking, (14) Being told you had a smoking-related illness, and (15) The coronavirus outbreak. Respondents were asked to rate each of these on a three-point scale with the options “not at all,” “somewhat,” or “very much,” but we dichotomized each so the last two options together were compared to “not at all” in the analyses.

### Analyses

All analyses were performed with SPSS version 27 and were weighted by age group, sex, region, and educational level to be representative of adult tobacco smokers in the Netherlands. We first examined differences between cis-women, cis-men, and gender minorities in demographic characteristics and the outcome variables with Chi-square tests. Subsequently, we used linear regression analyses (for the outcome Heaviness of Smoking Index and number of serious quit attempts) and logistic regression analyses (for all other outcome variables) with the three gender identity categories (cis-women, cis-men, and gender minorities) as independent variables, while adjusting for age group, educational level, and income level. Gender minorities were the reference category in these regression models. We then examined differences between cis-women and cis-men (excluding the gender minorities) on the outcome variables with both Chi-square tests and adjusted regression analyses. Cis-men were the reference category in the regression models. We also tested interactions between the gender identity categories by age group, educational level, and income level in separate models.

## Results

### Gender Minority Categorization

Respondents’ sex assigned at birth, current gender identity, and gender roles (mean scores on the TMF scale) are shown in Table 1. Most respondents who were categorized as gender minorities were individuals who scored around the midpoint of the TMF scale (neither masculine nor feminine, *n* = 203). Only a few of the respondents who were categorized as gender minorities identified as nonbinary, genderqueer, intersex, or a sex/gender not listed (*n* = 10). Others who were categorized as gender minorities had male sex assigned at birth and now identified as a woman (*n* = 3), had female sex assigned at birth and now identified as a man (*n* = 1), and had masculine gender roles while identifying as a woman or a female sex assigned at birth (*n* = 4), or had feminine gender roles while identifying as a man or a male sex assigned at birth (*n* = 11). There is some overlap between these responses, which can be seen in more detail in Table 1.

**Table 1.** Sex Assigned At Birth and Current Gender Identity By Gender Roles (the TMF Scale) (*n* = 1787)

Sex assigned at birth → ↓ Current gender identity	Female (*n*)	Male (*n*)	Intersex (*n*)	Sex not listed here (*n*)
TMF scale masculine (1–3)				
Woman (*n*)	2^c^	1^c^	0	0
Man (*n*)	1^c^	897^a^	1^c^	0
Nonbinary (*n*)	0	0	0	1^c^
Genderqueer (*n*)	0	0	0	0
A gender not listed here (*n*)	0	0	0	0
TMF scale midpoint (3.1–4.9)				
Woman (*n*)	117^c^	0	0	0
Man (*n*)	0	80^c^	0	1^c^
Nonbinary (*n*)	2^c^	0	1^c^	0
Genderqueer (*n*)	1^c^	0	0	0
A gender not listed here (*n*)	0	1^c^	0	0
TMF scale feminine (5–7)				
Woman (*n*)	670^b^	2^c^	0	0
Man (*n*)	0	7^c^	0	0
Nonbinary (*n*)	0	1^c^	0	0
Genderqueer (*n*)	0	1^c^	0	0
A gender not listed here (*n*)	0	0	0	0

TMF = Traditional Masculinity-Femininity.

^a^Cis-men.

^b^Cis-women.

^c^Gender minority populations.

### Differences in Demographic Characteristics

Table 2 shows differences in demographic characteristics between cis-women, cis-men, and gender minorities. There were significant differences in age groups between cis-women and gender minorities (*p* < .001) and between cis-men and gender minorities (*p* < .001). Gender minorities were more often 39–54 years old and less often 55 years and older compared to cis-women and cis-men. There was a significant difference in educational levels between gender minorities and cis-women (*p* < .001), gender minorities and cis-men (*p* = .034), and cis-women compared to cis-men (*p* = .021). Gender minorities were higher educated than cis-women and cis-men. Cis-men were higher educated than cis-women. Cis-men reported a significantly higher income level than cis-women (*p* < .001) and gender minorities (*p* < .001). Cis-women and gender minorities also differed significantly in their answers to the income question (*p* = .013), where the main difference between these groups was that cis-women more often did not report their income level.

**Table 2.** Differences Between Cis-women, Cis-men, and Gender Minorities on Demographic Characteristics

	Cis-women *n* (%)	Cis-men *n* (%)	Gender minorities *n* (%)	Cis-women compared to gender minorities	Cis-men compared to gender minorities	Cis-women compared to cis-men
Age groups						
18–24 years	103 (15.4)	123 (13.7)	33 (15.1)	**χ** ^**2**^ = **21.28**	**χ** ^**2**^ = **20.26**	χ^2^ = 7.33
25–39 years	156 (23.3)	264 (29.4)	63 (28.8)	***p* < .001**	***p* < .001**	*p* = .062
39–54 years	181 (27.0)	226 (25.1)	82 (37.4)			
55 years and older	230 (34.3)	286 (31.8)	41 (18.7)			
Educational level						
Low	292 (43.8)	338 (38.0)	65 (29.8)	**χ** ^**2**^ = **19.33**	**χ** ^**2**^ = **6.77**	**χ** ^**2**^ = **7.76**
Moderate	267 (40.0)	366 (41.1)	93 (42.7)	***p* < .001**	***p*** = **.034**	***p*** = **.021**
High	108 (16.2)	186 (20.9)	60 (27.5)			
Income level						
Low	257 (38.3)	214 (23.8)	97 (44.5)	**χ** ^**2**^ = **10.70**	**χ** ^**2**^ = **41.36**	**χ** ^**2**^ = **68.10**
Moderate	188 (28.0)	317 (35.3)	68 (31.2)	***p*** = **.013**	**p < .001**	***p* < .001**
High	56 (8.3)	175 (19.5)	21 (9.6)			
Not stated	170 (25.3)	192 (21.4)	32 (14.7)			

Bold values are significant at *p* < .05.

### Differences in Outcome Variables

There were no significant differences between cis-women, cis-men, and gender minorities in the heaviness of smoking, ever having tried to quit smoking, and the number of serious quit attempts (Supplementary Table 1). Plans to quit smoking differed between cis-men and gender minorities, but only in the unadjusted analysis (*p* = .016 vs. *p* = .077 in the adjusted analysis). Gender minorities were significantly more likely to have used cessation assistance in the past 6 months than cis-women and cis-men (*p* = .013 and *p* < .001 respectively, adjusted analyses).

Gender minorities were more likely to report the price of cigarettes (*p* = .031), concern for their personal health (*p* = .018), a smoking-related illness (*p* = .009), society’s disapproval of smoking (*p* = .029), advice from a doctor to quit (*p* < .001), free or lower cost stop-smoking medications (*p* = 0.033), warning labels on cigarette packages (*p* = .003), an anti-smoking message or campaign (*p* ≤ 0.001), the coronavirus outbreak (*p* < .001), and the availability of a telephone helpline (*p* = .001) as a trigger to think about quitting smoking than cis-men in the adjusted analyses. Additionally, in the unadjusted analyses, we found that gender minorities were more likely to report smoking restrictions at work as a trigger to think about quitting smoking than cis-men (*p* = .038).

Gender minorities were more likely to report advice from a doctor to quit (*p* = .002), an anti-smoking message or campaign (*p* = .001), smoking restrictions at work (*p* = .006), and the availability of a telephone helpline (*p* < .001) as a trigger to think about quitting smoking than cis-women in the adjusted analyses. Additionally, in the unadjusted analyses we found that gender minorities were more likely to report friends and family disapprove of smoking (*p* = .026), smoking restrictions at public places (*p* = .040), and warning labels on cigarette packages (*p* = .025) as triggers than cis-women.

There were also differences between cis-women and cis-men (Table 3). Cis-women were more likely to plan to quit smoking within the next 6 months than cis-men (*p* = .001, adjusted analysis). Cis-women were more likely to have used cessation assistance in the past 6 months than cis-men, but only in the unadjusted analysis (*p* = .046 vs. *p* = .072 in the adjusted analysis). Cis-women had also made a higher number of serious quit attempts in the past than cis-men (*p* = .067 unadjusted, *p* = .024 adjusted analyses). Cis-women were more likely to report the price of cigarettes (*p* = .025), society’s disapproval of smoking (*p* = .041), and the coronavirus outbreak (*p* = .004) as a trigger to think about quitting smoking than cis-men in the adjusted analyses. Additionally, in the unadjusted analyses, we found that cis-women were more likely to report a smoking-related illness (*p* = .014) and free or lower-cost stop-smoking medications (*p* = .026) as triggers than cis-men.

**Table 3.** Differences Between Cis-women and Cis-men on the Outcome Variables

	Cis-women (%)	Cis-men *n* (%)	Chi-square tests	Adjusted regression analyses (reference category = cis-men)
Heaviness of Smoking Index (HSI)^a^				
0 to 1	204 (30.8)	276 (31.4)	χ^2^ = 0.08, *p* = .961	ß = −0.02, *p* = .339
2 to 4	416 (62.8)	548 (62.4)		
5 to 6	42 (6.3)	54 (6.2)		
Plans to quit smoking within next 6 months (% yes)	200 (29.9)	216 (24.0)	**χ** ^**2**^ = **6.68, *p*** = **0.010**	**OR** = **1.48, *p*** = **.001**
Used any kind of cessation assistance in the past 6 months (% yes)	82 (12.2)	82 (9.1)	**χ** ^**2**^ = **3.99, *p*** = **0.046**	OR = 1.36, *p* = .072
Ever tried to quit smoking (% yes)	501 (74.8)	638 (71.0)	χ^2^ = 2.69, *p* = .101	OR = 1.22, *p* = .100
Number of serious quit attempts (among those who ever tried to quit)				
One attempt	88 (18.3)	149 (24.3)	χ^2^ = 7.15, *p* = .067	**ß** = **0.07, *p*** = **.024**
Two attempts	174 (36.3)	214 (34.9)		
Three attempts	97 (20.2)	125 (20.4)		
Four attempts or more	121 (25.2)	126 (20.5)		
Triggers for thinking about quitting smoking (% somewhat or very much)				
The price of cigarettes	519 (77.5)	638 (71.0)	**χ** ^**2**^ = **8.36, *p*** = **.004**	**OR** = **1.32, *p*** = **.025**
Concern for your personal health	434 (64.8)	562 (62.6)	χ^2^ = 0.80, *p* = .372	OR = 1.13, *p* = .269
Setting an example for children	367 (54.8)	480 (53.5)	χ^2^ = 0.27, *p* = .603	OR = 1.12, *p* = .281
Concern about the effect on nonsmokers	276 (41.2)	354 (39.4)	χ^2^ = 0.50, *p* = .479	OR = 1.06, *p* = .581
A smoking-related illness	271 (40.4)	309 (34.4)	**χ** ^**2**^ = **6.00, *p*** = **.014**	OR = 1.19, *p* = .120
Friends and family disapprove of smoking	255 (38.1)	363 (40.4)	χ^2^ = 0.86, *p* = .353	OR = 0.94, *p* = .593
Society’s disapproval of smoking	243 (36.3)	296 (32.9)	χ^2^ = 1.90, *p* = .168	**OR** = **1.26, *p*** = **.041**
Advice from a doctor to quit	216 (32.2)	250 (27.8)	χ^2^ = 3.61, *p* = .057	OR = 1.12, *p* = .336
Free/lower-cost stop-smoking medication	221 (33.0)	250 (27.8)	**χ** ^**2**^ = **4.98, *p* =.026**	OR = 1.21, *p* = .098
Smoking restrictions in public places	181 (27.0)	254 (28.3)	χ^2^ = 0.29, *p* = .588	OR = 0.89, *p* = .318
Warning labels on cigarette packages	171 (25.5)	205 (22.8)	χ^2^ = 1.53, *p* = .216	OR = 1.19, *p* = .154
An anti-smoking message or campaign	146 (21.8)	198 (22.0)	χ^2^ = 0.01, *p* = .912	OR = 0.99, *p* = .958
The coronavirus outbreak	130 (22.5)	122 (15.5)	**χ** ^**2**^ = **10.92, *p* < .001**	**OR** = **1.51, *p*** = **.004**
Smoking restrictions at work	134 (20.0)	211 (23.5)	χ^2^ = 2.73, *p* = .098	OR = 0.82, *p* = .113
Availability of telephone helpline	92 (13.7)	116 (12.9)	χ^2^ = 0.23, *p* = .632	OR = 0.96, *p* = .782

^a^HSI is categorized into three levels for this table; HSI was used as a continuous variable in the regression analyses (with higher values indicating greater dependence).

Bold values are significant at *p* < .05.

### Interactions

We also tested whether there were significant interactions of gender identity with age group, educational level, and income level for all of the outcome variables (not in tables).

Interaction analyses including cis-women, cis-men, and gender minorities showed significant interactions between gender identity and income level for plans to quit smoking (*p* of interaction = .015) and for use of cessation assistance (*p* = .033). Gender minorities with a low-income level were more likely to plan to quit smoking and were more likely to use cessation assistance than cis-women and cis-men. We also found interactions between gender identity and age for 3 triggers for thinking about quitting smoking (the availability of a telephone helpline, *p* = .033; friends and family disapproval of smoking, *p* = .011; a smoking-related illness, *p* = .025). Gender minorities who were younger were more likely to report the availability of a telephone helpline as a trigger than cis-women and cis-men and were more likely to report friends and family disapproval of smoking as a trigger than cis-women. Gender minorities who were older were more likely to report a smoking-related illness as a trigger than cis-men.

Interaction analyses including only cis-women and cis-men showed a significant interaction between gender identity and age for a smoking-related illness as a trigger to think about quitting smoking (*p* = .016). Older cis-women were more likely to report this trigger than older cis-men.

## Discussion

In this study, we examined differences between gender minority populations, cis-women, and cis-men in the heaviness of smoking, quit intentions, use of cessation assistance, quit attempts, and triggers for thinking about quitting smoking. We found that gender minority smokers in the Netherlands were significantly more likely to use cessation assistance than cis-women and cis-men. Gender minority smokers were also more likely to report several triggers for thinking about quitting smoking, for example, quit advice from a doctor, an anti-smoking message or campaign, and the availability of a telephone helpline. Gender minority populations did not differ from cis-women and cis-men in plans to quit smoking and quit attempts in adjusted analyses. This may mean that gender minority smokers are just as interested in quitting smoking as other smokers. Their higher use of cessation assistance combined with equal levels of the heaviness of smoking suggests that they are more responsive to assist with smoking cessation, but not necessarily because they are more nicotine-dependent. Part of the explanation could be that gender minority populations are more in need of cessation assistance because of stress, mental health problems, or a lack of social support.^31^ However, this does not provide a full explanation, as we also know that gender minority populations are less likely to seek and receive health care services in general.^32^ Therefore, this should be examined more specifically in future studies, for example with qualitative studies with which it is possible to gain a deeper understanding of individuals’ needs and motivations.

We also examined differences in smoking cessation between cis-women and cis-men. Cis-women were significantly more likely to plan to quit smoking within the next 6 months than cis-men, which is in line with previous research.^33,34^ Cis-women had also made a higher number of serious quit attempts in the past than cis-men. Cis-women were more likely to report the coronavirus outbreak as a trigger for thinking about quitting than cis-men. Other research from the Netherlands did not find a difference between women and men in their motivation to quit smoking due to the coronavirus.^35^ Differences in how the questions were formulated and when the data were collected may explain these differences. The data from Elling et al.^35^ were collected in March and April 2020, at the beginning of the COVID-19 pandemic. Data from our study were collected between September and November 2020, when people had more time to experience how the coronavirus actually changed their motivation to quit smoking and more scientific knowledge about the effects of the coronavirus on smokers was available. Cis-women were also more likely to report the price of cigarettes as a trigger for thinking about quitting than cis-men, which was also found in other countries.^36^

We explored more subgroup differences by examining interactions between gender identity and age group, educational level, and income level. Knowledge about subgroup differences in smoking cessation may help to create more effective targeted smoking cessation interventions. We found several significant interactions. Gender minorities with a low-income level were more likely to plan to quit smoking and were more likely to use cessation assistance than cis-women and cis-men with a low-income level, while we did not see these gender identity differences among respondents with higher income levels. Furthermore, gender identity differences in reporting trigger for thinking about quitting smoking differed between age groups. Gender minorities who were younger were more likely to report the availability of a telephone helpline and friends’ and family’s disapproval of smoking as a trigger for thinking about quitting smoking. Gender minorities who were older were more likely to report a smoking-related illness as a trigger than cis-men who were older, and cis-women who were older were also more likely to report this trigger than cis-men who were older. Besides these subgroup differences, it would be important to examine interactions with ethnic minority status in future research, as we know that intersecting minority statuses can cause additional stress for which smoking could be a coping method.^31^ We could not examine this with our sample because only a few respondents were part of an ethnic minority group.

### Implications for Practice

Gender minority populations in our sample used cessation assistance more than cis-women and cis-men. There is little evidence on whether smoking cessation programs are as effective among gender minority populations.^1,2^ Belonging to a minority population can trigger stress and mental health problems and smoking is often used as a coping mechanism against minority stress, which can make quitting even more difficult.^31,37^ Smoking cessation counselors should therefore be sensitive to the stressors that individuals from any minority population face, such as stigmatization, discrimination, and loneliness, and should educate their smoking clients on effective coping mechanisms to prevent relapse into smoking after they experience these stressors. Another solution would be to develop tailored smoking cessation programs or campaigns specifically for gender minority populations.^2,31^ Based on the results of our subgroup analyses, programs or campaigns for younger gender minority smokers could focus on the availability of telephone helplines and on how friends and family think about their smoking behavior.

### Strengths and Limitations

A strength of our study is that we examined differences between cis-women, cis-men, and gender minority populations. This last group is often omitted from health research, sometimes because researchers do not differentiate between sex assigned at birth and gender identity or gender roles, and sometimes because the group is too small for further analyses. For our study, we used a combination of sex assigned at birth, current gender identity, and gender roles to divide our sample into three groups. If we only would have used sex assigned at birth and current gender identity, then our gender minority group would have been too small for analyses in our study as well. Grouping the gender identity minority group and the gender role minority group together in our analyses is not the perfect solution, but a first step to explore this topic by using a population survey.

The TMF scale is a validated scale to measure gender roles with high reliability.^28^ We have used the scale to define gender roles in minority populations, which is a new way to use the scale and it has not been validated for this purpose. It is possible that through identifying individuals as being part of the gender minority population based on their gender roles in addition to their sex and gender identity, individuals have been categorized as gender minority while they do not identify as such. For example, individuals who do not agree with the traditional expected gender roles for their sex assigned at birth, but do not identify themselves as part of the gender minority population, instead identifying themselves as cis-women or cis-men. Although our choice can be justified on the basis that individuals with gender roles that do not match their sex assigned at birth or gender identity may experience gender role conflict,^8,29^ there are also validated scales available to measure gender role conflict which we did not use in our study.

Other limitations are that we used self-reported data and that we gave no definition in our survey of what qualifies as a serious quit attempt. Finally, a limitation that should be mentioned is that we used cross-sectional data for our study. Although this is not a problem for the independent variable of sex assigned at birth, we do not know when respondents formed their current gender identity or gender roles. As such, it is not appropriate to draw causal conclusions from this study.

### Conclusion

Gender minority populations in the Netherlands used cessation assistance more than cis-women and cis-men. Gender minority populations were also significantly more likely to report several triggers for thinking about quitting smoking. They did not differ from cis-women and cis-men in the heaviness of smoking, plans to quit smoking, and quit attempts. It is important that we not only examine the use of cessation assistance and triggers to think about quitting among gender minority populations, but that we also take it a step further by examining in future research how these individuals can effectively be helped to quit smoking.

## Supplementary Material

A Contributorship Form detailing each author’s specific involvement with this content, as well as any supplementary data, are available online at https://academic.oup.com/ntr.

ntac283_suppl_Supplementary_Table

## Data Availability

In each country participating in the international Tobacco Control Policy Evaluation (ITC) Project, the data are jointly owned by the lead researcher(s) in that country and the ITC Project at the University of Waterloo. Data from the ITC Project are available to approved researchers 2 years after the date of issuance of cleaned data sets by the ITC Data Management Centre. Researchers interested in using ITC data are required to apply for approval by submitting an International Tobacco Control Data Repository (ITCDR) request application and subsequently signing an ITCDR Data Usage Agreement. The criteria for data usage approval and the contents of the Data Usage Agreement are described online (http://www.itcproject.org).
