# Supplementary material for: Smoking Cessation Among Gender Minority Populations, Cis-women, and Cis-men: Findings From the International Tobacco Control Netherlands Survey
Source: Nicotine Tob Res. 2022 Dec 13;25(5):945–53. doi: 10.1093/ntr/ntac283 (PMC10077939; doi:10.1093/ntr/ntac283)
Supplement: ntac283_suppl_Supplementary_Table [file ntac283_suppl_supplementary_table.docx]

Supplementary Table 1: Differences between cis-women, cis-men, and gender minorities on the smoking related outcome variables.

|  | Cis-women  n (%) | Cis-men  n (%) | Gender minorities  n (%) | Cis-women compared to gender minorities | Cis-men compared to gender minorities |  | Cis-women compared to gender minorities | Cis-men compared to gender minorities |
| --- | --- | --- | --- | --- | --- | --- | --- | --- |
|  |  |  |  | Chi-square tests | |  | Adjusted regression analyses | |
| Heaviness of Smoking Index^a^ |  |  |  |  |  |  |  |  |
| 0 to 1 | 204 (30.8) | 276 (31.4) | 59 (27.3) | χ^2^=0.97 | χ^2^=1.44 |  | ß=-0.06 | ß=-0.04 |
| 2 to 4 | 416 (62.8) | 548 (62.4) | 142 (65.7) | p=0.615 | p=0.487 |  | p=0.088 | p=0.280 |
| 5 to 6 | 42 (6.3) | 54 (6.2) | 15 (6.9) |  |  |  |  |  |
| Plans to quit smoking within next 6 months (% yes) | 200 (29.9) | 216 (24.0) | 70 (32.0) | χ^2^=0.35  p=0.555 | **χ^2^=5.83**  **p=0.016** |  | OR=1.07  p=0.699 | OR=0.74  p=0.077 |
| Used any kind of cessation assistance in past 6 months (% yes) | 82 (12.2) | 82 (9.1) | 44 (20.1) | **χ^2^=8.37**  **p=0.004** | **χ^2^=21.19**  **p<0.001** |  | **OR=0.59**  **p=0.013** | **OR=0.45**  **p<0.001** |
| Ever tried to quit smoking (% yes) | 501 (74.8) | 638 (71.0) | 168 (77.1) | χ^2^=0.46  p=0.496 | χ^2^=3.17  p=0.075 |  | OR=0.87  p=0.449 | OR=0.72  p=0.071 |
| Number of serious quit attempts (among those who ever tried to quit) |  |  |  |  |  |  |  |  |
| One attempt | 88 (18.3) | 149 (24.3) | 29 (17.7) | χ^2^=0.06 | χ^2^=4.06 |  | ß=0.00 | ß=-0.08 |
| Two attempts | 174 (36.3) | 214 (34.9) | 59 (36.0) | p=0.997 | p=0.255 |  | p=0.942 | p=0.088 |
| Three attempts | 97 (20.2) | 125 (20.4) | 34 (20.7) |  |  |  |  |  |
| Four attempts or more | 121 (25.2) | 126 (20.5) | 42 (25.6) |  |  |  |  |  |
| Triggers for thinking about quitting smoking (% somewhat or very much) |  |  |  |  |  |  |  |  |
| The price of cigarettes | 519 (77.5) | 638 (71.0) | 177 (81.2) | χ^2^=1.35  p=0.245 | **χ^2^=9.30**  **p=0.002** |  | OR=0.88  p=0.524 | **OR=0.66**  **p=0.031** |
| Concern for your personal health | 434 (64.8) | 562 (62.6) | 156 (71.2) | χ^2^=3.08  p=0.079 | **χ^2^=5.74**  **p=0.017** |  | OR=0.75  p=0.095 | **OR=0.67**  **p=0.018** |
| Setting an example for children | 367 (54.8) | 480 (53.5) | 116 (53.2) | χ^2^=0.16  p=0.687 | χ^2^=0.00  p=0.949 |  | OR=1.10  p=0.545 | OR=0.98  p=0.906 |
| Concern about the effect on non-smokers | 276 (41.2) | 354 (39.4) | 101 (46.1) | χ^2^=1.64  p=0.200 | χ^2^=3.27  p=0.070 |  | OR=0.81  p=0.178 | OR=0.76  p=0.074 |
| A smoking-related illness | 271 (40.4) | 309 (34.4) | 96 (44.0) | χ^2^=0.87  p=0.350 | **χ^2^=7.03**  **p=0.008** |  | OR=0.79  p=0.137 | **OR=0.66**  **p=0.009** |
| Friends and family disapprove smoking | 255 (38.1) | 363 (40.4) | 102 (46.6) | **χ^2^=4.98**  **p=0.026** | χ^2^=2.78  p=0.095 |  | OR=0.75  p=0.065 | OR=0.78  p=0.113 |
| Society's disapproval of smoking | 243 (36.3) | 296 (32.9) | 91 (41.6) | χ^2^=1.97  p=0.161 | **χ^2^=5.79**  **p=0.016** |  | OR=0.89  p=0.458 | **OR=0.71**  **p=0.029** |
| Advice from a doctor to quit | 216 (32.2) | 250 (27.8) | 87 (39.7) | **χ^2^=4.12**  **p=0.042** | **χ^2^=11.88**  **p<0.001** |  | **OR=0.60**  **p=0.002** | **OR=0.54**  **p<0.001** |
| Free / lower cost stop-smoking medication | 221 (33.0) | 250 (27.8) | 76 (34.7) | χ^2^=0.21  p=0.650 | **χ^2^=4.05**  **p=0.044** |  | OR=0.84  p=0.312 | **OR=0.70**  **p=0.033** |
| Smoking restrictions in public places | 181 (27.0) | 254 (28.3) | 75 (34.2) | **χ^2^=4.21**  **p=0.040** | χ^2^=3.05  p=0.081 |  | OR=0.73  p=0.065 | OR=0.81  p=0.198 |
| Warning labels on cigarette packages | 171 (25.5) | 205 (22.8) | 73 (33.3) | **χ^2^=5.06**  **p=0.025** | **χ^2^=10.39**  **p=0.001** |  | OR=0.73  p=0.067 | **OR=0.61**  **p=0.003** |
| An anti-smoking message or campaign | 146 (21.8) | 198 (22.0) | 72 (32.9) | **χ^2^=10.96**  **p<0.001** | **χ^2^=11.32**  **p<0.001** |  | **OR=0.56**  **p=0.001** | **OR=0.56**  **p<0.001** |
| The coronavirus outbreak | 130 (22.5) | 122 (15.5) | 53 (29.0) | χ^2^=3.14  p=0.076 | **χ^2^=18.19**  **p<0.001** |  | OR=0.78  p=0.198 | **OR=0.52**  **p<0.001** |
| Smoking restrictions at work | 134 (20.0) | 211 (23.5) | 66 (30.3) | **χ^2^=9.95**  **p=0.002** | **χ^2^=4.32**  **p=0.038** |  | **OR=0.61**  **p=0.006** | OR=0.75  p=0.092 |
| Availability of telephone helpline | 92 (13.7) | 116 (12.9) | 52 (23.7) | **χ^2^=12.19**  **p<0.001** | **χ^2^=16.21**  **p<0.001** |  | **OR=0.51**  **p<0.001** | **OR=0.53**  **p=0.001** |

^a^ HSI is categorized in three levels for this table; HSI was used as a continuous variable in the regression analyses (with higher values indicating greater dependence).
Bold values are significant at p<0.05.
